# Descriptive understanding and prediction in COVID-19 modelling

**DOI:** 10.1007/s40656-021-00461-z

**Published:** 2021-09-21

**Authors:** Johannes Findl, Javier Suárez

**Affiliations:** 1 LOGOS/BIAP, Department of Philosophy, Facultat de Filosofia, Univerity of Barcelona, C/ Montalegre 6-8, Room 4049, 08001 Barcelona, Spain; 2 Department of Philosophy of the Natural Sciences, Institute of Philosophy, Jagiellonian University of Krakow, Grodka 52, Room 42, 33-332 Krakow, Poland

**Keywords:** SARS-CoV-2, Description, Scientific explanation, Epidemiological modelling, Statistical modelling

## Abstract

COVID-19 has substantially affected our lives during 2020. Since its beginning, several epidemiological models have been developed to investigate the specific dynamics of the disease. Early COVID-19 epidemiological models were purely statistical, based on a curve-fitting approach, and did not include causal knowledge about the disease. Yet, these models had predictive capacity; thus they were used to ground important political decisions, in virtue of the understanding of the dynamics of the pandemic that they offered. This raises a philosophical question about how purely statistical models can yield understanding, and if so, what the relationship between prediction and understanding in these models is. Drawing on the model that was developed by the Institute of Health Metrics and Evaluation, we argue that early epidemiological models yielded a modality of understanding that we call *descriptive understanding*, which contrasts with the so-called *explanatory understanding* which is assumed to be the main form of scientific understanding. We spell out the exact details of how descriptive understanding works, and efficiently yields understanding of the phenomena. Finally, we vindicate the necessity of studying other modalities of understanding that go beyond the conventionally assumed explanatory understanding.

## Introduction

COVID-19 was first reported on 31st December 2019 as a pneumonia of unknown aetiology that was observed in the Chinese province of Hubei.^1^ The first cluster was identified in the proximities of the Wuhan market, which was closed for disinfection on the 1st of January 2020. COVID-19 cases started to increase exponentially, quickly spreading to other parts of China. Less than two weeks later, on the 12th of January, the virus SARS-CoV-2 was identified as the causative agent of the disease, and data on its genomic composition was published for the first time. On the 13th of January, Thailand recorded the first case outside the geographic borders of China. By the end of January, reported cases of COVID-19 amounted to 7818, by which time the disease had been identified in 18 countries around the world, including cases in the USA and Canada, as well as in Germany, France, and Finland. By that time, Hubei had already been confined, with severe travel restrictions imposed. The main fear was that at the observed speed at which the infection rate was growing, healthcare systems would soon become overwhelmed in those areas particularly affected by COVID-19. That fear had become a reality in Wuhan, where the army was required to set up a campaign hospital with a capacity for 1,000 patients that began operating on the 3rd of February. The disease continued to spread around the globe, with the World Health Organization (WHO) declaring it a pandemic on the 11th of March 2020. Less than a week later, on the 17th of March, most European countries imposed severe restrictions on their citizens’ basic rights by declaring nationwide lockdowns—shutting down non-essential businesses, issuing stay-at-home orders and closing borders. Such countermeasures required most countries to impose a state of emergency.^2^

The situation was critical in several European countries, as was reflected in data about the occupancy of intense care units (ICUs), the observed collapse of emergency services in hospital, and the resulting need to build field hospitals for basic assistance in several countries.^3^^,^^4^ By March 2020, little was known about the nature of SARS-CoV-2 and, specifically, little was known about how it could spread so quickly. Importantly, the political decision makers who adopted countermeasures heavily relied upon epidemiological models that predicted how the virus would spread, and how it would stop spreading under certain restrictions. Early versions of such models based their predictions on statistical data that had been provided by other countries, rather than on a causal understanding of the disease. In other words, early COVID-19 models were what epidemiologists call *statistical models*, i.e., models that derive their estimations from a regression analysis that fits a curve to empirical data—such as the number of infections or deaths—rather than from causal data about the patterns of infection of the disease which were mostly unknown at the time.^5^

Hence, political decision making was informed by estimations derived from *purely predictive* epidemiological models.^6^ While these models did not include specific causal-mechanistic information about how the disease would spread or affect those infected, their primary function was to give estimates of what would most likely happen if counter-measures were introduced or removed; for instance, how long would it take for the rate of infections to decrease and how this would affect hospital occupation. Furthermore, these models were built and modified according to the observed effects of countermeasures in other parts of the world (e.g., how the restrictions imposed in Wuhan changed the local infection and mortality rate), yet these modifications were vastly contingent upon the observed data in certain locations, without tracking why the data differed in this particular way. In this sense, statistical models became the main tool to gain knowledge about the dynamics of the COVID-19 pandemic from its early stages onwards.

From a philosophical perspective, this form of modelling also raises an interesting question about the relationship between the scientific capacity to predict a phenomenon and the ability to understand it; a topic that had already stimulated the interest of philosophers (de Regt, 2017; Dieguez, 2013; Douglas, 2009; Elgin, 2017; Frigg & Hartmann, 2020; Potochnik, 2017), and scientists (Shmueli, 2010).^7^ Since early epidemiological models were tested as to whether their predictions fit the reported data, it was possible to discover which of the underlying model assumptions were incorrect, which ones were lacking, and which ones had a different effect than had initially been assumed. Doing so, in turn, led to the development of new and more precise versions of statistical models. This development suggests that gradually, epidemiologists acquired a better understanding of the main variables determining the trajectory of the death rate than the one they had at the beginning of the pandemic. Yet, this acquisition was possible even in the absence of an explanation of the exact relationship between COVID-19 and the accompanying mortality and infection rates. For this reason, we believe that by studying the development of one of these models in its detail, we will be in a good position to analyse how the concepts of prediction and understanding are related to each other. We aim to spell out the nature of this relationship in some detail by looking at how understanding emerges in a specific case study (see Poliseli, 2020 for a similar approach).

We will focus on the development of the model from the Institute of Health Metrics and Evaluation (IHME model), and carefully analyse how it was modified in the light of new evidence. Concretely, we analyse the role that early predictions played, and how their comparison with the evidence ultimately resulted in a modified model with a better data-fit which was not based on the knowledge of an explanation—*explanatory understanding*—of the phenomenon. In view of this, we argue that early IHME predictions generated a specific type of understanding—which we call *descriptive understanding*, or *DESC*—of the relationship between certain restrictions and the evolution of the infection rate. As a result, this descriptive understanding was used to predict the evolution of hospital occupation, which served politicians as a basis to impose or relax restrictive measures.

Overall, our paper shows that in the early IHME COVID-19 epidemiological model, prediction and understanding are in an intimate dialectical relationship that is not mediated by an explanation, but by a description. Our observation is at odds with views that define understanding as consisting in *having an explanation* (de Regt, 2017; Hills, 2016; Khalifa, 2017). In contrast, our case study favours those accounts according to which understanding is a very specific *skill* of the members of a scientific community that can be realised through a plurality of cognitive pathways (Dellsén, 2020; Verreault-Julien, 2019).

In Sect. 2, we show how other analyses of the relationship between prediction and understanding presuppose that an explanation is always mediating between both elements. In Sect. 3, we present the IHME model and argue that it serves as a fruitful case study to study the interplay between prediction and understanding, due to its own development during the COVID-19 pandemic. In Sect. 4, we show that in the IHME model, prediction and understanding are not mediated by an explanation, but by a different type of cognitive path. In Sect. 5, we introduce the concept of *descriptive understanding* as the type of understanding that emerges in the building-process of the early versions of the IHME model. We further show the epistemological relevance of descriptive understanding, showing the role that predictions play in creating and improving it. Finally, we present our conclusions.

## Understanding, explanation, and prediction

Several philosophers have alluded to the existence of a relationship between prediction and understanding in scientific modelling (Elgin, 2017; Khalifa, 2017; Potochnik, 2017), though many of them have failed to spell out clearly what this relationship exactly amounts to. In some cases, this has been due to the lack of a precise account about the exact epistemological mediation between both concepts. In other cases, while the exact epistemological mediator is clear—an explanation—the cognitive path between the two concepts is not, leaving open the conceptual possibility that other types of cognitive mediators exist.

Grimm (2010) and Hills (2016) present an example of the first problem. In analysing what an agent’s understanding consists of, these authors have argued that it is a cognitive ability (often called *grasping*) that enables one “to draw the conclusion that *p* (or probably *p*) from the information that *q*” (Hills, 2016), or, less formally, to “anticipate how changes in one element of the thing under consideration will (or will not) bring about changes in another element of the thing.” (Grimm, 2010, p. 342). Assuming that prediction can be at least minimally conceived as a form of inference, hence equated to something along the lines of “drawing a conclusion from a body of information or evidence” or “anticipating consecutive changes between elements”, it follows that Grimm and Hills consider that understanding, as a cognitive ability, makes prediction feasible. What is more, according to these authors, understanding is manifested in the capacity of the subject(s) that possess it to generate predictions from the body of knowledge that is available to her. This results in the view that understanding and prediction are epistemically and, probably, semantically, connected. Unfortunately, neither Grimm nor Hills say more about what this connection exactly consists of, nor how both concepts constitutively assist each other in scientific research. Is prediction strictly necessary for understanding, or is it just a way, among many others, of manifesting it? Understanding may make predictions feasible but, are there other ways of doing so, when understanding is not present? Moreover, how tight is the relationship between both concepts?

On the other hand, a good example of the second problem can be perceived in the work of Douglas (2009). Focusing on how *explanatory* models and theories provide understanding of some phenomena, she has suggested that predictions play the epistemically crucial role of testing explanations in so far as they “assist our explanatory endeavors by providing a check on our imagination, helping to narrow the explanatory options to those that will provide a more reliable basis for decision making” (Douglas, 2009, p. 446). According to this view, explanations are a key epistemological concept mediating between prediction and understanding, as understanding is equated to *having an explanation*. This results in a model according to which predictions enhance our understanding by telling us which of our explanations are the correct ones, and which are not. This view enriches Grimm’s and Hills’ accounts epistemologically, but it is not particularly informative of the cognitive path from explanation to prediction and back. Are predictions *the only test* between alternative explanations? If not, then it seems the relationship between understanding and prediction is seriously weakened. If they were *the only test*, then it would be necessary to say what the appeal to explanation as a mediating concept is exactly adding to the specification of the relationship between prediction and understanding.

A more informative, yet we think problematic account of the relationship between prediction and understanding is provided by de Regt (De Regt, 2009, 2017; de Regt & Dieks, 2005), who builds on Douglas’ framework but also spells out its details in considerable depth. We consider that his view of the connection is the most articulated so far; thus, we will concentrate on his analysis here. However, as we will show, his method of analysing the relationship is slightly problematic in accounting for our case study (Sect. 3) insofar as de Regt takes explanation as *the* epistemological concept mediating between prediction and understanding. To see why, let us first introduce de Regt’s analysis of scientific understanding, as well as his terminology.

In chapter 4 of his book *Understanding Scientific Understanding*, de Regt introduces the two key notions that structure his contextual approach to the concept of scientific understanding, namely the *Criterion for Understanding Phenomena* (what we have so far called “explanation”; hereafter, CUP) and the *Criterion for the Intelligibility of Theories* (what we have called “understanding” so far; hereafter, CIT). These two criteria are explained as follows:

CUP: A phenomenon P is understood scientifically if and only if there is an explanation of P that is based on an intelligible theory T and conforms to the basic epistemic values of empirical adequacy and internal consistency (de Regt, 2017, p. 92)

CIT: A scientific theory (T) (…) is intelligible for scientists (in context C) if they can recognize qualitatively characteristic consequences of T without performing exact calculations (de Regt, 2017, p. 102)^8^

In de Regt’s work, these two criteria are associated with a definition of intelligibility (understanding, in our terminology) as the value that scientists ascribe to the set of characteristics of a scientific theory that facilitates its use (in making models, providing explanations, etc.) (de Regt, 2017, p. 23). As this set of characteristics is contextual (viz. it may change from one research community to another), and relational (viz. it depends on the skills of the scientists who use these theories), it is impossible to specify a set of necessary and sufficient characteristics that makes a theory intelligible. In any case, the concept of intelligibility, and its connection with CUP and CIT, provides a first link between the concepts of understanding and prediction. De Regt tells us: “The intelligibility of a theory (…) implies that it should be possible to grasp how its predictions are generated” (2017, p. 102). Given that scientists are the ones who build the models, construct the explanations, and are ultimately responsible for the predictions, it can be argued that the former sentence entails that if a theory is intelligible to a scientist, then she can derive predictions from it. This reading suggests that having intelligible theories^9^ is *sufficient* for making predictions. Call this feature the *prediction-generating character* of intelligible theories. The key question now is whether, and if so, how, it is possible to make a conceptual move from the prediction-generating character of intelligible theories to their ability to provide explanatory scientific understanding of certain phenomena (i.e., to CUP).

**Fig. 1:**
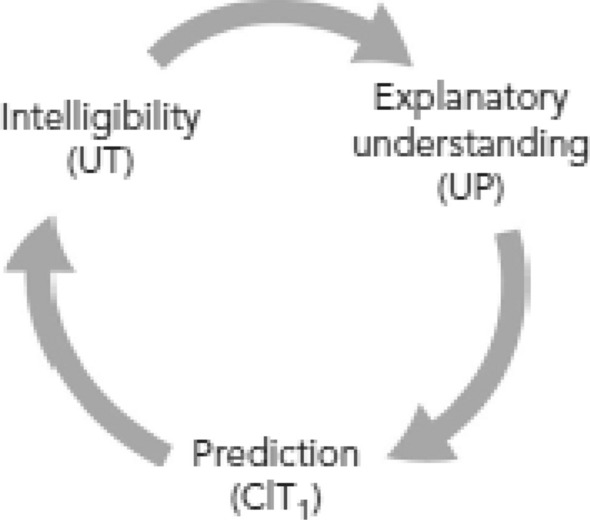
Analysis of the interplay between prediction, explanatory understanding, and intelligibility in de Regt’s model of understanding. Note that explanations are always in between intelligibility and prediction (de Regt, 2017, p. 108, Fig. 4.1)

Let us assume, for simplicity, that we have a prediction-generating theory which is also empirically adequate and internally consistent. Does this entail that the theory provides understanding of some phenomena? The key to answer this question lies in the connection between explanation and prediction. Given that an empirically adequate and internally consistent theory is one that produces explanations, de Regt’s theory of understanding can answer our question affirmatively, for he assumes the existence of an “inherent connection between prediction and explanatory understanding” (2017, p. 107).This assertion is essential, for it proves that in de Regt’s conception of scientific understanding, scientific explanations *always* play a mediating role between mere intelligibility and predictions. This is analogous to Douglas’ idea, whom de Regt appeals to when articulating the nature of this relationship: “the relation between explanation and prediction is a tight, functional one: explanations provide *the cognitive path to* predictions, which then *serve to test and refine* the explanations” Douglas (2009, p. 454, emphasis added). De Regt’s conceptual map of the connections between explanation, understanding and prediction can be seen in Fig. 1.

This reading of the connection between prediction and understanding presents two problems. Firstly, as it happened with Douglas’ account, it is not clear what the exact “cognitive paths” from explanations to prediction are, or in which way predictions “serve to test and refine” explanations. Are the cognitive paths mathematical calculations? Or psychological operations? How do predictions refine explanations? By proving them false? On the other hand, what de Regt’s figure suggests is that there is a conceptual move from explanation to prediction, and from the latter to intelligibility, which then goes back to the idea of explanation. But what type of conceptual move? Is it a question of pragmatics or does it have an epistemological import? If it has an epistemological import, how does it specifically work? De Regt neither elaborates concrete examples, nor does he spell out possible characteristics. Thus, so formulated, the connection remains vague, and in need of further research that investigates its precise nature.

Secondly, the reading of de Regt’s work we have just suggested seems to be at odds with other assertions that he makes in *Understanding Scientific Understanding*. Remember that the primary reading we have offered casts intelligible theories as sufficient for generating predictions. However, CIT casts the relationship as *necessary*. Namely, it asserts that scientists can recognise qualitatively characteristic consequences of a theory (i.e., predictions; see de Regt, 2017, p. 107) only if it is intelligible to them. This reading is reasonable (i.e., not contradictory) for, if one looks at Fig. 1, it is obvious that the conceptual move goes from explanatory understanding, to prediction, and later to intelligibility. However, The problem is that de Regt’s theory assumes, correctly in our view, a circularity or, as we prefer to express it, a dialectical relationship that develops with time. How this relationship develops, and whether it necessarily requires an explanation in between intelligibility and prediction, is precisely the question we are investigating in this paper.

The necessary reading of the relationship is furthermore consistent, although not equivalent, with his assertion that “prediction turns out to be impossible without understanding” (2017, p. 107). The concept of impossibility puts us in the realm of modality. If a modal reading is adopted, then *we must renounce the possibility of predictive, non-explanatory modelling practices that provide understanding*. De Regt believes that this possibility should be rejected as a case of non-genuine understanding:

Perhaps it is possible to devise a purely phenomenological model of a phenomenon, which does not relate to any theories at all, *but such a model would merely have a descriptive and perhaps predictive value but yield not explanatory understanding* (2017, p. 98, emphasis added)

The intuition according to which no understanding is possible without an explanation is reinforced later. De Regt tells the imaginary story of an oracle that produces perfect predictions of every phenomenon, and wonders whether scientists would be satisfied with such a perfect tool. He says:

An oracle is nothing but a black box that produces seemingly arbitrary predictions. Scientists want more than this: in addition they want insight, and therefore they need to open the black box and consider the workings of the theory that generates the predictions (2017, pp. 101–102)

**Fig. 2:**
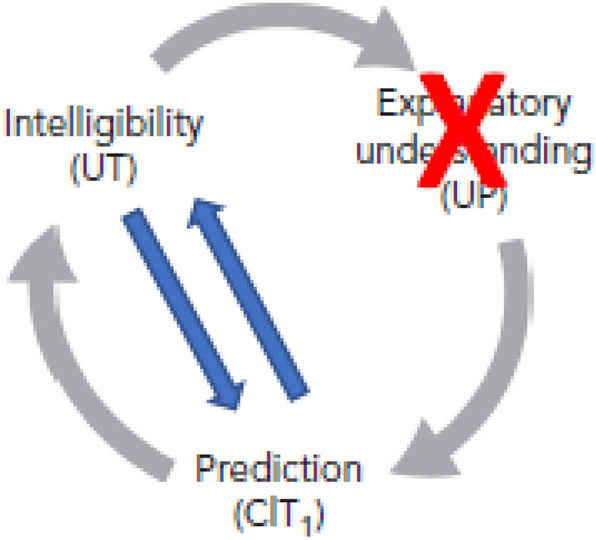
We investigate whether there is a connection between understanding (intelligibility) and prediction that does not require an explanatory step, as well as the exact details of how this relationship works

Even while he rejects that such an oracle is realisable in our world, it is beyond doubt that the intuition of the oracle is a conceptual possibility that requires it to be taken seriously. Concretely, because the possibility of an oracle suggests that non-explanatory understanding may exist and maybe also plays a role in science. In other words, because the possibility of imagining such an oracle makes it feasible to imagine a *prediction-generating* theory/model/scientific tool that provides understanding but does not simultaneously generate explanations (Fig. 2). We show an example of such a possibility, spelling out the details of the relationship, including the cognitive path between understanding and prediction, in the remainder of this paper (Sects. 4, 5).

## Modelling COVID-19: An introduction to the Institute of Health Metrics and Evaluation Model

**Fig. 3:**
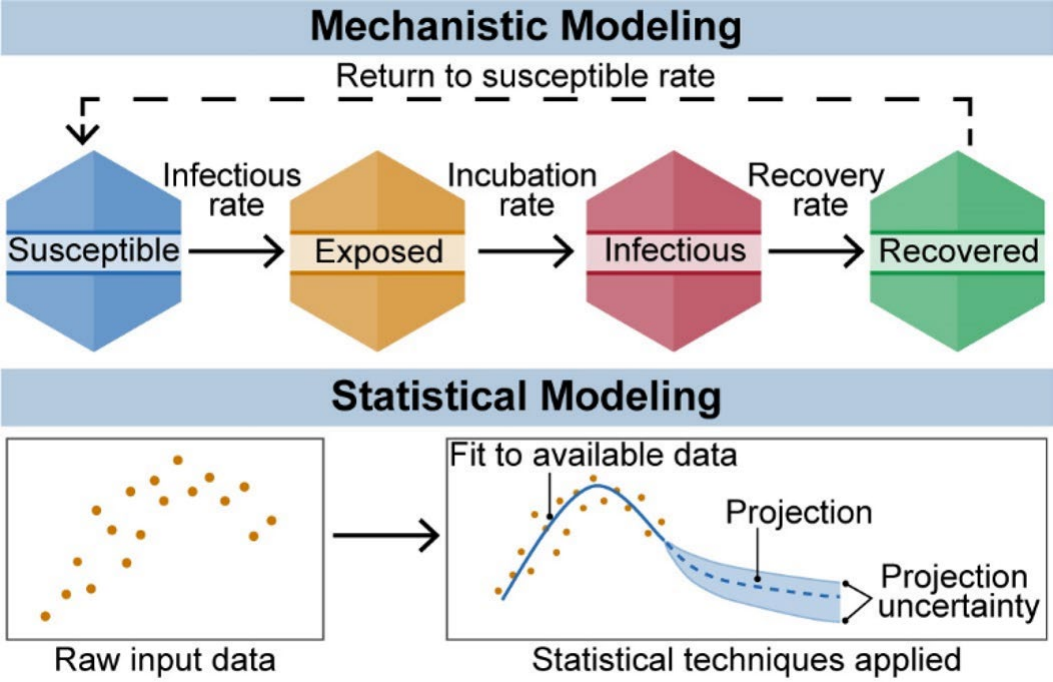
There are two general types of infectious disease models: mechanistic models, which use causal knowledge of disease transmission and dynamics, and statistical models, whose predictions rely only on patterns in the data (GAO, 2020)

Since the beginning of the COVID-19 pandemic, research groups from all over the world have built epidemiological models to estimate the impact of the outbreak of the disease, which in turn have been heavily relied upon by policymakers in their decision-making. COVID-19 models can be broadly distinguished by three different types: *statistical models* that derive their estimations from a regression analysis that fits a curve to empirical data such as the number of infections or deaths, *mechanistic models* that simulate disease transmission between (groups of) people on the basis of empirical data such as the spread of the virus, the onset of disease symptoms, and *hybrid models* that combine both approaches (Fig. 3).

While the choice for one type of model can be explained by the different purposes for which it was designed, such as estimating the disease’s short-term impact versus investigating future scenarios (Maziarz & Zach, 2020), the accuracy of the output, i.e., the estimates of all types of models, depends on the quality of the data to which models are calibrated, which at an early stage of an epidemic is typically limited. Limitations for statistical COVID-19 models have been attributed to insufficient or inconsistent detection of cases or, delays in reporting or errors in documentation, whereas limitations for mechanistic COVID-19 models have stemmed from insufficient understanding of the biological nature and behaviour of the virus.

In the course of the pandemic, COVID-19 models have been constantly fed with new data, modified by smoothing the available data and refined by tweaking their parameters, some of which have also been substantially updated by extending the underlying regression algorithm or, in some cases, by adding a mechanistic component to the model.

To be clear, even in their more advanced versions, COVID-19 models should not be thought of as “crystal balls”, as Michael Levy (2020) has recently remarked. While we will show that the evolution of models such as the IHME model has given rise to improvements in their predictions, we can never reasonably expect them to give predictions as precise as the ones we can find in the physical sciences. As any model needs to rely on a limited set of parameters, with human behaviour thought to be too complex to be expressed by such a set, the best we can hope for is that its predictions do not diverge too far from reality and be accurate enough to inform decision-making; a demand which in light of the COVID-19 pandemic, threatening the lives of many and impacting the worldwide economy in an unprecedented way, becomes particularly important.

What exactly determines the degree of permissible divergence and what makes an estimate sufficiently accurate are of course delicate questions. Answering them in a fully satisfactory way may well lie beyond the scope of this paper. Suffice it to say that it is nevertheless possible to identify instances of clearly impermissible divergence and insufficiently accurate estimates, such as the IHME model’s severe underprojection of the number of total deaths in the US, which has arguably led to a delay in the adoption of COVID-19 control measures such as social distancing and closure of schools there, which, if it had been left uncorrected, would have resulted in their premature relaxation.

### The model of the University of Washington’s Institute for Health Metrics and Evaluation (IHME Model) in detail

The model of the University of Washington’s Institute for Health Metrics and Evaluation (IHME) was one of the most prominent statistical COVID-19 models that was used early on in the COVID-19 pandemic (first released on March 26, 2020) to estimate the death rate and the excess demand for beds and ventilators in hospitals at its estimated peak. The first version found, at a very general level, that even with the enactment of social distancing measures, the epidemic would place a load on health systems beyond available capacity.

The majority of early COVID-19 forecasts was based on mechanistic models that predicted disease transmission on the basis of estimating the probability of people moving between susceptible, exposed, and infected states, and then to a recovered state or death (SEIR models). These SEIR models generally suggested that most, if not all, individuals in a population would become infected unless countermeasures were introduced and therefore projected millions of deaths from COVID-19 in the USA and Europe. Most SEIR models could not predict peaks and subsequent declines in deaths and, importantly, were not able to account for the effects that individual behavioural responses and government-mandated countermeasures could have on the course of the epidemic (IHME, 2020).

In light of these shortcomings, the IHME scientists decided for the alternative strategy of statistically modelling the empirically observed COVID-19 population death rate curves (for further motivations with regard to this choice, see Sect. 4) The statistical approach of the early IHME model consisted of two basic components. The first was a nonlinear mixed effects regression framework that projected the course (viz. the trajectories of the cumulative and daily death rate) of the epidemic by trying to fit a specific sigmoid function—a Gaussian error function–to the shape of the epidemic as a function of the implementation of social distancing measures. The corresponding bell curve depicted the number of deaths rising and falling and finds where US data of confirmed deaths fit on that curve. The second component of the model was a microsimulation that estimates the need for hospitalisation, ICU use, and ventilation based on available data on clinical practices in COVID-19 patients (Murray et al., 2020). Let us now consider the model’s first basic component (viz. its nonlinear mixed effects regression framework) in more detail.

The first step in developing the basic component was to compare different functional forms for modelling the death rate of COVID-19 and see how they fit to the available data. The IHME scientists found that a Gaussian Error Function provided the best fit and developed a statistical curve-fitting tool which they called *CurveFit* on its basis (IHME, 2020). Hence, one key assumption underlying this modelling approach was that the cumulative death rate for each location would follow the parametrised Gaussian Error Function in Eq. 1:

1 $$ D(t;\alpha ,\beta ,p) = \frac{p}{2}\Psi \left( {\alpha (t - \beta )} \right) = \frac{p}{2}\left( {1 + \frac{2}{\sqrt \pi }\int_{0}^{{\alpha \left( {t - \beta } \right)}} {\exp \left( { - \tau^{2} } \right)} d\tau } \right) $$

Note that the Gaussian Error function in Eq. 1 has three fundamental parameters α, β, p that can be fit to data (viz. confirmed COVID-19 deaths), where α represents mortality growth, β the timing of when the growth curve inflects, and p the final total. As the parameters by themselves do not account for the covariates reflecting social distancing measures, statistical models are used to specify these through link functions, thereby connecting different locations together, and through fixed and random effects. In the first version of the model, these priors were chosen by determining the mean variance of the relationship between the social distancing covariates and the peak time from Wuhan City, China, where the only complete pandemic had been observed at that time. Finally, the result is a nonlinear mixed effects model with user-specified priors in the statistical assumptions (IHME, 2020).

The reason for choosing confirmed deaths rather than reported cases of infection was that IHME researchers thought the former to be more accurately reported than the latter, especially in the beginning of the pandemic.

Soon after its release, the model came under heavy attack for the large discrepancy between estimated and actual deaths, having severely underprojected the death toll. For example, while the 27 March version had projected that NY would at most see 26,444 deaths (i.e., the upper range of its estimates), with an estimated median of 10,243 deaths, 31,125 people had actually died by June 22. Moreover, it was found that the actual number of deaths fell outside the IHME’s next day 95 percent confidence interval 70 percent of the time (Marchant et al., 2020).

**Fig. 4:**
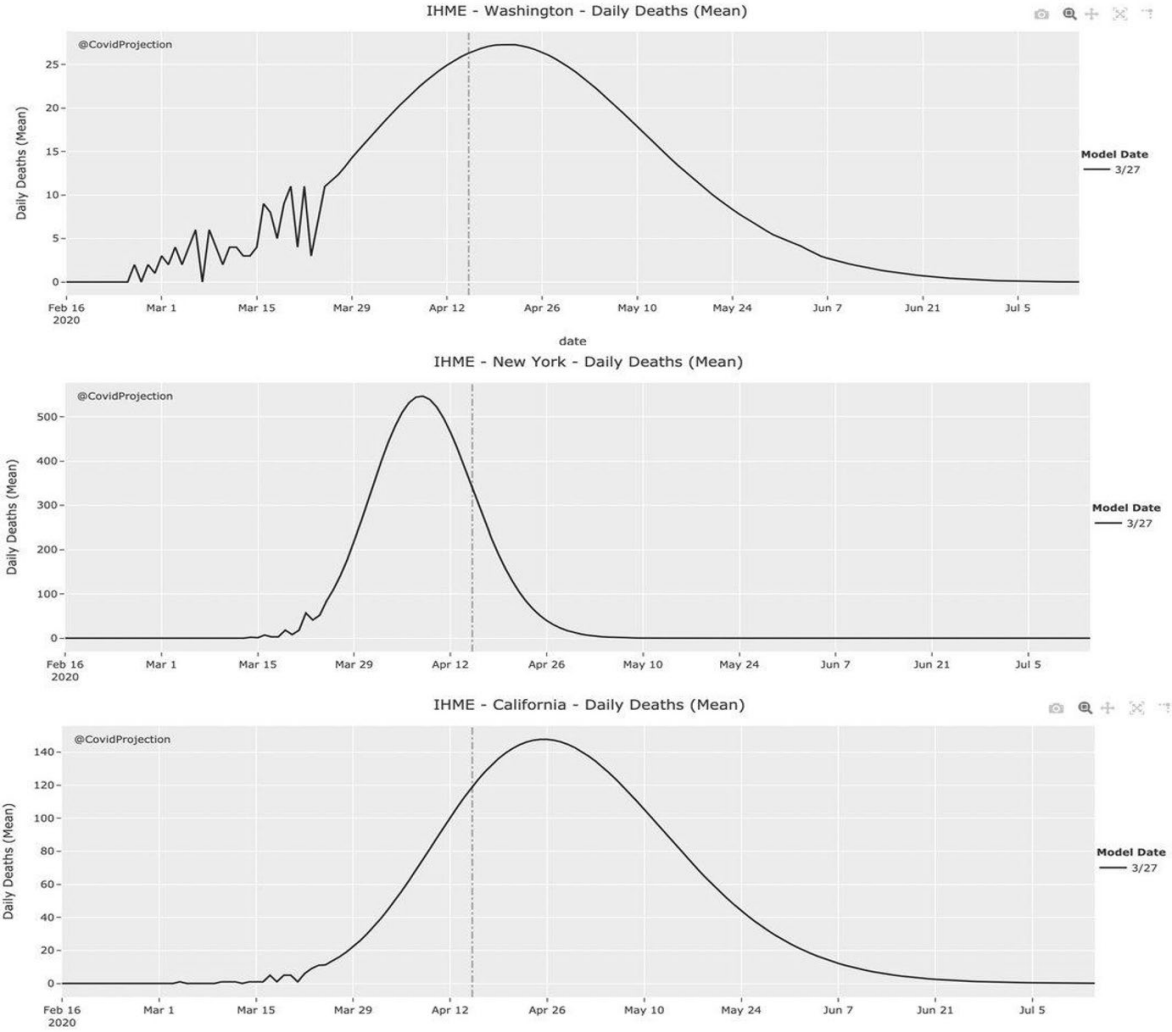
The early IHME model predicted COVID-19 curves for Washington, New York, and California that appear highly symmetric, which is (COVID-projections) the result of fitting a Gaussian error function to the data representing the cumulative number of deaths that have occurred

The severe failure of the IHME model, especially in its early versions, has been mainly attributed both to the fact that it is a purely statistical model that does not account for transmission dynamics (Jewell et al., 2020) and to the very choice of the Gaussian error function (Bergstrom, 2020), which produces trajectories that appear highly symmetric, meaning that the pandemic is projected to rise to its peak and decline from it at the same rate. This can be seen in Fig. 4, which shows IHME’s projections as of 27 March for Washington, New York, and California.

**Fig. 5:**
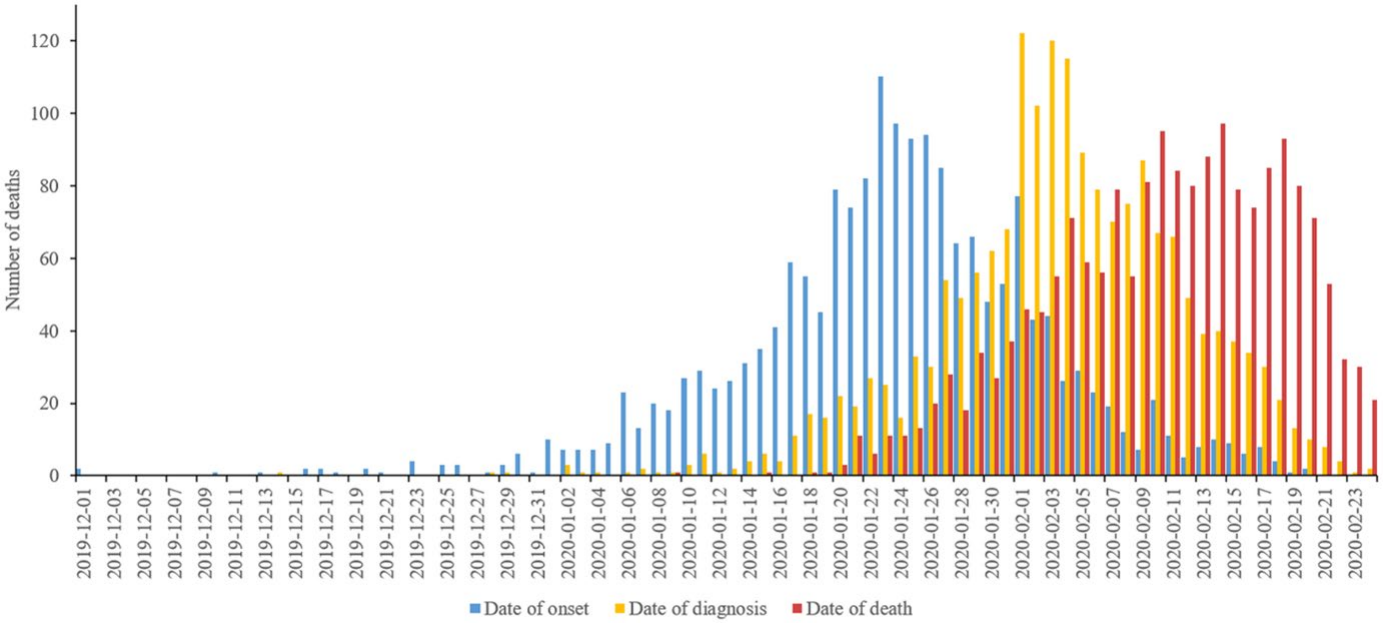
Epidemiological curves by date of symptom onset, date of diagnosis and date of death in Wuhan (Bai et al., 2020)

Choosing an epidemiological function that assumes symmetric bell curves to enable the estimation of the relevant parameters is historically motivated by the so-called Farr Law of Epidemics (Pacheco-Barrios et al., 2020), a single mathematical formula which tried to capture the bell-shaped curve that had been empirically observed in many epidemics, such as the Great Plague in London and Newcastle upon Tyne (Dean et al., 2018). Importantly, a similar, roughly symmetric shape has also been observed for the COVID-19 pandemic in Wuhan City in February 2020 (Fig. 5).

**Fig. 6:**
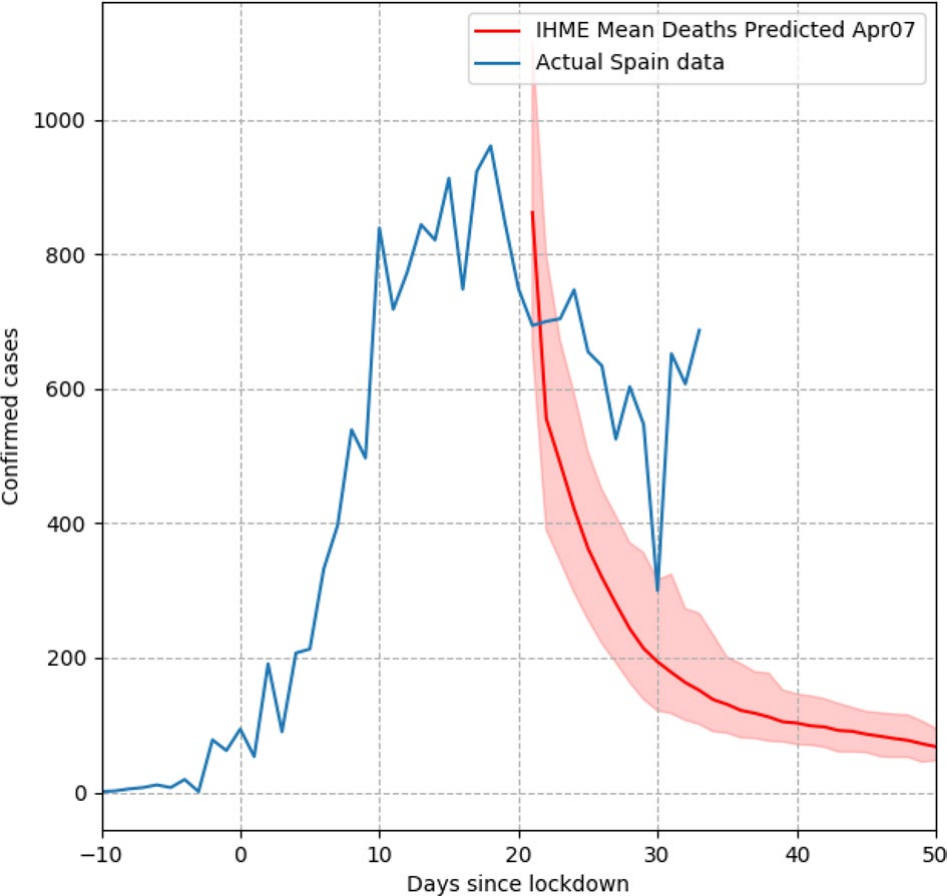
The actually observed death rate largely diverged from the one projected by an early IHME model (Tyka, 2020)

However, the assumption that the curves for the US states and for other countries would also follow a symmetric trajectory with an equal growth and decline rate was disconfirmed by empirical observations from several locations where the pandemic had peaked. These showed declines that took longer than was estimated by the model, which in turn underestimated the total number of deaths. The actually observed curves were characterised by a flat peak as can be seen in the example of Spain (Fig. 6).

In light of the shortcomings of its initial versions, the IHME model has received several updates, out of which two can be considered particularly important, with the first being designed to accommodate asymmetric bell curves, and the second to model people’s behaviour under an increasing variety of social distancing measures. While the latter would merit an analysis of its own, for reasons of scope, in what follows we will only consider the first update.

### April 17 update

To accommodate the observed asymmetries, the April 17 update (IHME, 2020) introduced a multiple mixture model component (“Gaussian extensions”). Roughly, such extensions can be understood as an extended approach to fit to data, using a linear combination of peaks inferred from different locations, in order to provide a weighting scheme that is then applied to new locations, i.e., locations that have not yet peaked.

In the first step, a particular basic model is fit to a given location using the social distancing covariate to fit its γ multiplier, and the parameters α and p, thereby inferring a peak. This gives the atom specification for the next step (the so-called “Gaussian atom”). For the specified atom, the IHME researchers then use a semi-parametric fit of staggered atoms to data, which means that they repeat the procedure from the first step until 12 further atoms are obtained from locations where a peak has been observed. They then consider a basis of staggered atoms for 13 days, with peaks 2 days apart, which are then centered at the inferred peak from step 1. Given a set of atomic functions of time fi(t), and all observations y_t_ for a given location, the following model is fit to data:

$$ y_{t} = \sum\limits_{i = 1}^{13} {w_{i} f_{i} (t) + \varepsilon } . $$

Since the data is fit as a non-negative combination of atoms, upper bound constraints of 1 are placed on each weight. The full fitting problem is a bound-constrained linear least squares problem which is solved by the L-BFGS-B routine (IHME, 2020):

2 $$ \mathop {\min }\limits_{{\left\{ {0 \le w_{i} \le 1} \right\}}} \sum\limits_{t} {\left( {y_{t} - \sum\limits_{i = 1}^{13} {w_{i} f_{i} (t)} } \right)}^{2} . $$

**Fig. 7:**
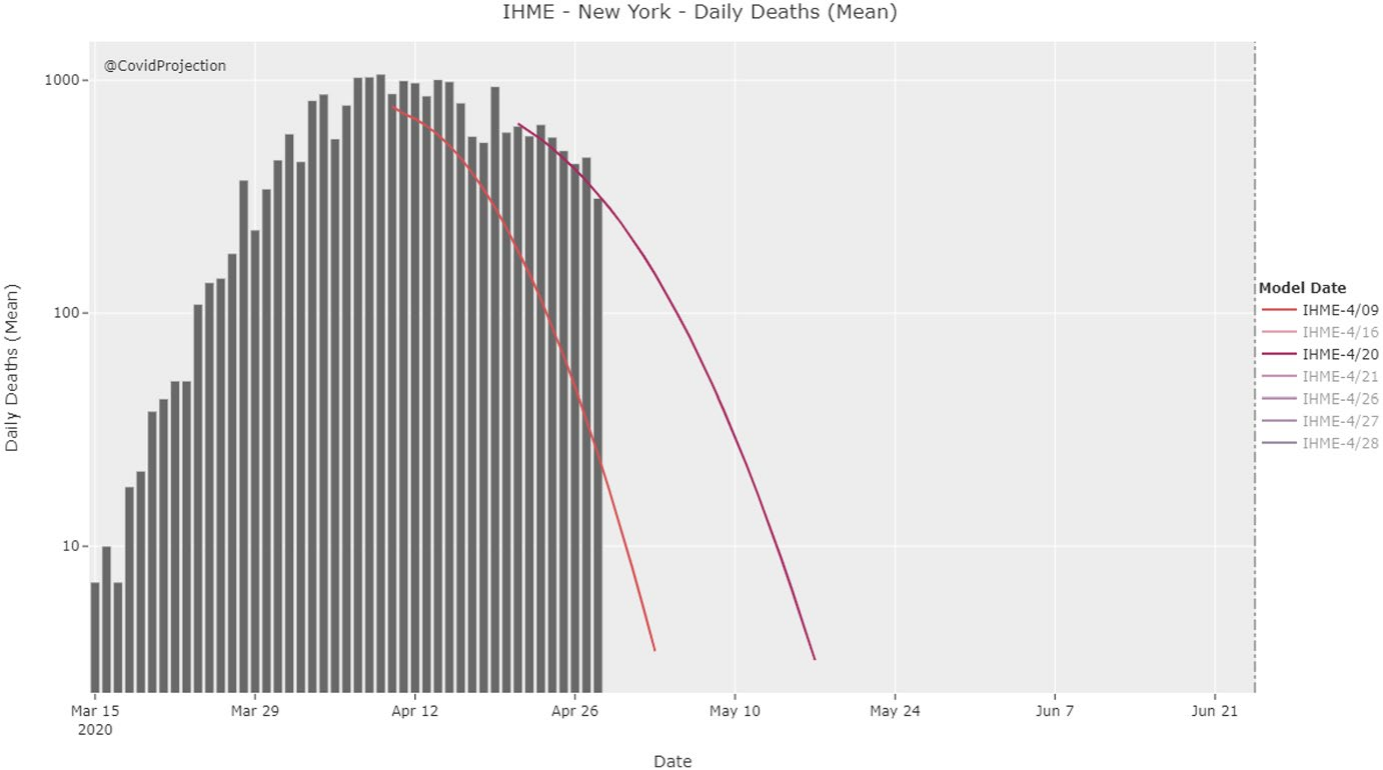
The actual number of deaths between the end of April and the beginning of May, represented by the blocks, was tracked much better by the updated model (Bergstrom, 2020)

The introduction of the weighting scheme finally allowed the IHME model to accommodate for asymmetric curves, which constituted the most important extension since its release, as was confirmed by the leading IHME scientist Chris Murray (FiveThirtyEight 2020). Through this update, the model could significantly increase the accuracy of its estimates, i.e., projecting a higher number of cumulative deaths because of a slower decline in the daily death rate. Figure 7 illustrates the sharp contrast between the predictions offered by the original IHME model and the version obtained after the April 17 update.

## Prediction and understanding without an explanation

As we said in Sect. 1, the development of the IHME model since the beginning of the COVID-19 pandemic provides a fruitful source for analysing the interplay between prediction and understanding in contemporary scientific practice.^10^ More concretely, our analysis aims to show two things. First, that the IHME model satisfies de Regt’s intelligibility requirement (i.e., it provides understanding according to our terminology) and does so via its predictions; second, that no explanation mediates between intelligibility and predictions (as so-called explanatory understanding would have it), but rather *descriptions* do. By what we will call *descriptive understanding* or *DESC*, we introduce a new modality of understanding not previously appreciated in philosophical debates. This section analyses why the IHME model is intelligible in de Regt’s sense (i.e., why it generates understanding), and why the type of predictions it generates cannot be considered the result of explanatory understanding. Section 5 will argue that the IHME provides descriptive, as opposed to explanatory understanding.

Let us start with the claim that the IHME model is intelligible in de Regt’s sense. Recall that intelligibility is a value that scientists in a particular context attribute to those qualities of their theory or model which facilitate its use. This claim thus depends on whether the IHME epidemiologists used a model-building strategy which would make their model intelligible in a way that facilitated its use at the beginning of the pandemic, as well as in its further development. The first versions of the IHME model followed a curve-fitting approach, which was pragmatically justified as the IHME epidemiologists had previously been using it to predict health-related outcomes in several epidemic scenarios long before COVID-19. Therefore, they already knew how to use the curve-fitting approach to predict the course of disease outbreaks. Moreover, and not less importantly, the scientists strongly believed *on evidential grounds* that a curve-fitting approach would be a superior choice to the mechanical approaches that other modellers, such as the Imperial College COVID-19 response team, had chosen. They believed that a problem with mechanical approaches in the early stages of a disease is that they would necessarily be built upon several assumptions about disease spread that would not be specific to COVID-19, but rather extrapolated directly from the observed behaviour of other viruses. The usefulness of the model would thus be limited to the contingency that COVID-19 mechanically behaves as other infectious diseases do; an assumption which in the beginning of COVID-19 was purely speculative. This clearly limits their validity and usefulness in early stages of an emerging pandemic caused by the unknown pathogen SARS-CoV-2. The IHME epidemiologists thought that a model following a curve-fitting approach would be more promising, and developed more easily because its predictions would be primarily informed by the empirical knowledge specific to the transmission rate of COVID-19 available at that time (Wuhan), with the possibility of further extending the model to include new data (Sect. 5). As the director of IHME, Christopher Murray, reports:

Whereas a lot of the modelling groups are using very theoretical models [mechanistic models], we are trying to fit a model to the data we’ve seen already in the world and when it comes to infectious diseases, this matters a lot because all these infectious disease transmission models show exponential growth up to the point where everybody gets infected, but that’s not what we saw in Wuhan (…). Introducing social distancing really puts the brakes on transmission, so you get a much earlier peak and that was the critical thing we were trying to predict—when will the peak be because that’s what hospitals need to plan. If you want to think about it, fundamentally, what we’re capturing is the human behavioural response to the world around us. [By contrast], the models that the Imperial College and others have are essentially assuming that people are going to live their lives and not change anything that they do.^11^

In sum, the IHME scientists attributed the value of intelligibility to their model’s predictions precisely because these facilitated the model’s use, and the model’s development in the light of new data. They believed their model could render good predictive estimates of the peaks in COVID-19’s mortality rate because, unlike other approaches, the curve-fitting approach included the effects of social distancing measures empirically observed in Wuhan. Additionally, because their model was responsive to new evidence, it could be re-used and re-adapted when new data became available, which is an epistemic virtue that epidemiologists particularly value especially in the early moments of an emerging disease.

As the IHME model’s intelligibility is primarily ascribed to its predictive capacity, a common assumption among philosophers would be that a form of explanatory understanding must be mediating between them, as explanations lay down the fundaments for predictions (Sect. 2). However, this is not the case in the early versions of the IHME model, given the specific details of their curve-fitting approach. To be clear, we claim that the analysis of these early versions reveals that there is a specific type of cognitive path to understanding (*descriptive understanding*) that is characteristic of scientific work: descriptions. Importantly, this type of cognitive path does not qualify as an explanation in any non-trivial sense. To see why, let us examine how the IHME model was built.

The first, curve-fitting, version of the IHME model was built to reflect the *regularity pattern* that the COVID-19-derived mortality rate was expected to follow. To do so, the model was built upon certain assumptions concerning the *shape* of the curve of mortality. These assumptions, directly derived from the evidence coming from Wuhan, are intentionally included in the IHME model to generate a regularity pattern that can embed the data-model (i.e., the mortality rates that are observed in reality). In general, obtaining a regularity pattern is considered a virtue of bona fide scientific explanations ever since Hempel’s deductive-nomological/inductive-statistical account (e.g., Díez, 2014; Hempel, 1965; Woodward, 2019), and it is also considered a virtue under an unificationist lens (Kitcher, 1989). Additionally, causalist approaches to explanation also emphasise that explanations are based on the possibility of obtaining a causal regularity pattern that connects explanans and explanandum in an asymmetric manner (Salmon, 1984; Woodward & Woodward, 2003). Nonetheless, the types of counterexamples raised against Hempel’s model (symmetries, irrelevancies, etc.; see Salmon, 1989; Woodward, 2019) suggest that while obtaining a regularity pattern and including it in the explanans is necessary for providing an explanation, it is by no means sufficient. Additional requirements need to be satisfied concerning the way in which explanans and explanandum relate to each other that, we contend, in the case of the IHME model, were not fulfilled. Hence, looking at the regularity pattern *only* would mask the real explanatory/non-explanatory import of the model. A more detailed analysis of the assumptions is required.

In choosing the assumptions of the model, the IHME scientists selected a set of variables that they assumed could exert relevant causal influence on the evolution of the pandemic. These included two beliefs: a) that social distancing measures had a strong effect on the mortality rate, as they had empirically observed in Wuhan; and b) that the effect of the social distancing measures on the mortality rate would be very similar also for other locations. Both assumptions were neither trivial, nor unjustifiably speculative, since they had been borne out by previous pandemics—thus their alleged causal influence on the evolution of COVID-19. From the viewpoint of the IHME researchers, it seemed reasonable to believe that similar political restrictions on social movements would have the same effects on the mortality rate in different parts of the world. And *a fortiori*, the observation that epidemic events rise and fall in a roughly symmetric pattern had found expression in the so-called Farr-Law (Dean et al., 2018). Hence, it was by no means unreasonable to assume that COVID-19 could follow a similar trajectory.

However, basing the model upon these assumptions does not mean that the IHME model was a causal model, because these assumptions are not related to the causal underpinnings of COVID-19 in terms of SARS-CoV-2’s behaviour, something that causalist philosophers would demand of a bona fide scientific explanation (Machamer et al., 2000; Glennan, 2002; Bechtel & Abrahamsen, 2005; Craver, 2009; Kaplan & Craver, 2011). Kaplan & Craver spell out this requirement as follows:

In successful explanatory models (…) (a) the variables in the model correspond to components, activities, properties, and organizational features of the target mechanism that produces, maintains, or underlies the phenomenon, and (b) the (perhaps mathematical) dependencies posited among these variables in the model correspond to the (perhaps quantifiable) causal relations among the components of the target mechanism. (2011, p. 611)

By contrast, the early IHME model was built upon the empirical observations of the mortality rate observed in a specific location, and how this rate changed when certain restrictive measures were introduced, plus the general assumption that the correlations between *social distancing measures* and the *mortality rate* would work in the same manner in the rest of the world. Nothing in the way these assumptions were chosen, or the way the mathematical model was built (including the meaning of the variables) reflects a causal relationship between the form of the model and the mechanistic biology of SARS-CoV-2. It is hard to see how this would even remotely qualify as a mechanistic explanation of *the development of the mortality rate*. Our case would be analogous to the scientific use of Kepler’s laws. These laws are usually regarded as *descriptive* rather than *explanatory* because they account for the positions of the planets in terms of previously observed positions and the phenomenological pattern that could be deduced from fitting this data into a mathematical equation. It is undeniable that Kepler’s work constitutes a great achievement in the development of physics, because knowing the pattern of a specific phenomenon provides a lot of information about it. The same is true for the first version of the IHME model: knowing the pattern of the mortality rate is helpful not only scientifically, but also politically, as it helps in decision making. Yet one should not conflate *scientific achievement* with *scientific explanation*, because the latter is only a very specific form that the former can take.

The fact that the IHME model is itself non-causal in the sense developed by causalist philosophers does not necessarily mean, though, that it may not capture counterfactual dependencies of a certain kind. Some recent approaches to scientific explanation have shown that certain types of scientific activities can provide bona fide explanations based on the existence of non-causal forms of counter-factuality (Baker, 2009; Díez, 2014; Moreno & Suárez, 2020; Rice, 2015; Strevens, 2008). Probably the most salient examples in contemporary science are topological explanations (Deulofeu et al., 2021; Huneman, 2010, 2018; Kostić, 2020; Suárez & Deulofeu, 2019). In a sense, it seems plausible to assume that the assumptions upon which the IHME model is built capture certain counterfactual dependencies between the social distancing measures and the mortality rate. But this is again incorrect, because it would attribute to the assumptions a role that they ultimately lack. The assumptions are not in any significant sense *within* the model, as if the IHME model were capturing the dependency relationships between social distancing measures and the mortality rate. Recall Murray’s interview (see above): the assumptions rather justify the choice of the curve-fitting approach, as opposed to other types of modelling approaches, including those chosen by competing groups. The regularity pattern generated by the model is not per se counterfactual. While it offers location-to-location variation, especially after the April update (Sect. 3.1), those within-model variations only reflect the evolution of the mortality rate in certain locations, and how their patterns matched ordiffered from those of Wuhan. It is assumed, for good reasons as we will show (Sect. 5), that these variations are due to the different local effects of the social measures. This hardly makes the case for an explanatory role of the assumptions, even in a weak counterfactual sense.

## Introducing descriptive understanding: from understanding to prediction via a description

So far, we have shown that the type of understanding gained in the process of building the curve-fitting versions of the IHME model (including the March version and the April update) is not explanatory. Nevertheless, a question remains about what type of understanding the building-process of the IHME model provides, and what it consists of. In this section, we argue that the early versions (including the April update) of the IHME model provided *descriptive understanding*, which is enough to generate predictions about the phenomenon which are scientifically useful insofar as they can be compared with the real mortality data, despite being a non-counterfactual model. Descriptive understanding can be characterised as follows:

**DESC**: A scientific community has descriptive understanding of a phenomenon P when they have a model or theory that can generate non-counterfactual predictions of the dynamics that P will follow (i.e., how the values of P will develop over time) and is built on a set of basic empirically-based assumptions A_1_, A_2_, A_3_,, …, that make these predictions plausible.

Note that *DESC* comes in degrees for, as we will show, the more adequate the set of assumptions that justifies the plausibility of the process of generating non-counterfactual predictions, the higher the degree of the *DESC* of a scientific community. The remainder of this section analyses the interplay between *DESC* and the production of predictions about the empirical phenomenon in the case of the IHME model. We show that *DESC* dynamically emerges and improves during this process.

To generate the first predictions with the early versions of their model, the IHME scientists started by choosing *a technical framework* that, in light of the two key assumptions they had made concerning the general evolution of pandemic, was adequate to model the mortality rate over time. The key assumptions were a) that the inclusion of social distancing measures in different locations will influence the evolution of the mortality rate, and b) that these effects will be similar for any location, i.e., that similar social-distancing measures will affect the evolution of the mortality rate in the same way irrespectively of the location. In the case of this specific pandemic, b) means that the curve of the mortality rate will always have a symmetric shape for every location, given that this was the shape observed in Wuhan (Sect. 3). For the technical framework, the epidemiologists decided to choose a Gaussian error function, based on their observation of how the COVID-19 mortality rate had already evolved in Wuhan, and based on previous experience with the behaviour of other disease outbreaks. The Gaussian error function for COVID-19 corresponds to a curve-fitting approach, i.e., a model that aims to fit the type of fluctuations in daily mortality rates observed in a few locations. Moreover, the model went slightly further in advancing some key predictions as to how the data would evolve in the following days, given what was known by the time the model was built. Interestingly, these predictions were not merely the result of a projection of the model into the future, following an arithmetic or geometric progression. Rather, the predictions were based on the integration between the model as described in Sect. 3, plus the fundamental assumptions a) and b).

**Fig. 8:**
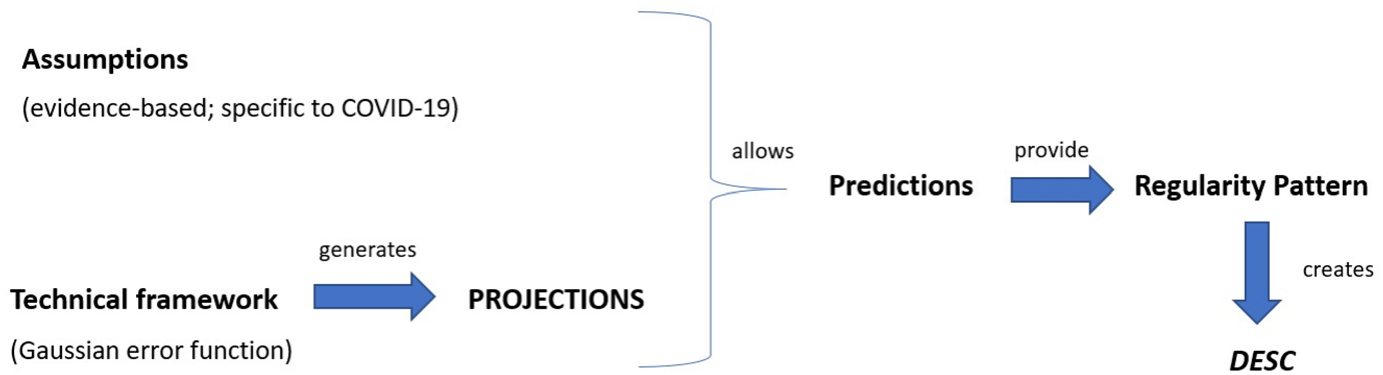
Schematic representation of the process that allows the first degrees of *DESC* to be obtained. Deepening *DESC* requires comparison of the values in the model’s regularity pattern with the real data

The integration of these two epistemic elements allowed the creation of a model, (Eq. 1) which in turn made possible the generation of a set of non-trivial predictions—as opposed to *projections*, which would be generated by the technical framework without the inclusion of any assumption in its building process—that jointly provided a regularity pattern of the expected mortality rate which was supposed to be numerically similar to the pattern that would emerge from observing real mortality data in different locations. It is in the very process of creating this model, we argue, that the scientists started to obtain *DESC*. Figure 8 schematically illustrates this claim. The key elements that characterise the model building process in which *DESC* can be obtained are the following: (a) the *technical framework*, as represented by the Gaussian error function; (b) the *assumptions* which are used to determine the shape of the Gaussian error function and allow for its precise mathematical expression; (c) the *regularity pattern* that the combination of (a) and (b) as expressed in the IHME model is expected to follow.

The interplay between prediction and *DESC* in this case works as follows. First, the technical framework produces basic “predictions” in the form of a *mere* (geometric) *projection* of the mortality rate into the future. Importantly, this technical framework needs to be adequate (*intelligible*) for modelling a specific pandemic like COVID-19. Yet, by itself, it is a mere predictor-generator which provides no understanding (i.e., neither in the explanatory, nor in the DESC mode) of the COVID-19 mortality rate. Second, the technical framework is combined with some fundamental assumptions about how the mortality rate generally behaves, and, as a result of this integration, a clear mathematical expression of the model follows. This expression allows the derivation of predictions that, according to the IHME epidemiologists (Sect. 4), make descriptive understanding of the mortality rates feasible. This is so because these predictions are plausible for the specific disease due to the empirical appeal of the initial assumptions given the evidence *available at the time when the first version of the model was built*.

While the empirical appeal of the assumptions is necessary for the emergence of *DESC*, it is by itself not sufficient. An additional necessary ingredient as to why the epidemiologists began to obtain *DESC* on the basis of these predictions is because they *convey a regularity pattern specific to the COVID-19 mortality rate that can then be compared to an empirically observed rate*. The regularity pattern is based on the evidence available at the time when the model is built and will allow (by means of its comparison to real data) for specific improvements in the model in its later versions. This last element is essential for the concept of *DESC* that characterises the work of the epidemiologists. Being conscious that a first model is only an approximation of the actual rates, they already work on the plausible assumption that some elements of the model will have to change in future versions due to the discovery of new evidence.

This gives us a picture according to which the model’s regularity pattern is intimately related to the two assumptions a) and b) about how the mortality rate will behave, since the pattern can only be generated with a technical framework that is integrated with assumptions a) and b), so that it can be universally applied. Furthermore, this picture points towards the existence of a deep, intimate relationship between *DESC* and prediction. But, importantly, it also points towards the relevance of combining specific assumptions about the disease under investigation with a technical framework, in a way which generates a non-trivial model that can generate regularity patterns, with future versions of the model being expected to generate better patterns that will give rise to an even deeper *DESC* (i.e., in virtue of further assessing the plausibility of the basic assumptions).

One may argue, though, that the process we have just spelled out either fails to characterize a genuine form of understanding—in the end, the model described by Eq. 1 had to be adjusted—or that it characterizes a rather shallow kind of understanding. In our specific case, the worry is that because the model simply follows the past behaviour of the COVID-19 mortality rate in some locations and projects it to other locations in the future, the IHME model would not allow for understanding *why* the rate behaves as it does. For example, by comparing the mortality rate observed in Spain with the rate projected by the early IHME model for Spain, one would understand *that* the real rate did not evolve symmetrically, but not understand *why* this was the case. So, how *DESC* is gained constitutes at best an uninteresting part of scientific research, and at worst not even a scientific achievement at all.^12^

The problem with this criticism is that what we have called *DESC* exists in several scientific areas and is essential for taking scientifically based political decisions, like e.g., in economics (Maziarz, 2020). Furthermore, *DESC* is even indispensable when carrying out research in contexts where causal knowledge is either not (yet) available or not considered useful for predictive or even practical purposes. For example, newly emerging disease outbreaks are typically due to unknown causes and gaining *DESC* of these diseases is essential for properly managing the disease.^13^ Additionally, causal models seem to be uninteresting for generating good predictions about the types of complications that may emerge after surgery, so that clinical surgeons tend to prefer descriptive models, and hence DESC, over gaining causal knowledge (Bernard, 2017). So, even if one may think that DESC is a *shallow* form of understanding, it is undeniable that it is a form of understanding that deserves philosophical scrutiny to better capture its epistemological import.

Second, while it is true that if the form of understanding we describe were exclusively gained by comparing the model’s projections with actual observations, it would be of a rather shallow nature, *DESC* is a far more complex kind of understanding that cannot be appreciated by *simply looking at the outcome of the modelling process*. While looking at the outcome of the modelling process may be useful to discover explanatory understanding, given it is based on the existence of counterfactual dependencies between the variables of the model, DESC does not emerge as a result or product of modelling, but rather as result of the process of model-building and model-readjusting in the light of new evidence. It is a form of understanding that can only be grasped by looking *at the development of the model* and *how epidemiologists changed it over time.*

While the case of *DESC* we have characterized in this paper corresponds to the form of understanding that emerges in the process of mathematical-model building, we suspect that an analogy with other processes of description would help to see the point we are making.^14^ Imagine we are trying to make a good description of the physiology of the nervous system, and we are supporting it with anatomical drawings of the neurons and the neural connections, including detailed knowledge of the different parts in the synaptic space. We can draft a first approximation of the drawing by looking at a series of samples, as Ramón y Cajal did. But the very first drawing will probably not be accurate enough for the purposes of capturing the details of the synaptic space, and once we have drawn it, it will become necessary that we look at the samples again and adjust these parts of our picture that do not correspond to the information that our samples reveal about the synaptic space. The process will need to be repeated several times, until our picture of the neural space is accurate and corresponds to what we aim at capturing based on what our samples reveal. *DESC* is what results from this whole process of drawing, redrawing, and comparing with the samples, until our picture is accurate and satisfactory. The final picture that we draw, i.e., the result of the process, cannot be characterized as providing us with *DESC*. Rather, in our account, *DESC* is what gradually emerges throughout the process.

Our argument is that the development of the IHME model constitutes a clear case of *DESC*, one that is obtained through mathematical modelling and, especially, *through model improvement*. It is this process of model-development what creates *DESC*, and not the final model itself. Let us now show how this process took place in the case of the IHME model.

As we already noted, the IHME model underwent an important alteration on April 17, when a multiple mixture model component was introduced to cope with the observed mismatch between the early predictions and the empirical data. Basically, the key problem of the first version of the IHME model was that the mortality rate developed very differently across the locations where measures were introduced. Consequently, the projected death rates diverged largely from the observed number of deaths, showing that the model had significantly underestimated the consequences of COVID-19. This led epidemiologists to revise the original assumptions that had backed up their early predictions and investigate which of them might have caused the mismatch. They found that their key assumption b), about the symmetrical behaviour of the mortality rate across locations, was mistaken for most local conditions. Note that, as we showed (Sect. 3), the symmetry of mortality curves is a well-established epidemiological observation (viz. the Farr Law of Epidemics) which had also been detected for the city of Wuhan. Epidemiologists worked on the assumption that if symmetry had been observed in the mortality rate-curve of Wuhan, then it was also expected to be shown in other locations. However, this assumption was proven false: observed death trajectories were characterised by a long tail which represented a slow decline of deaths, resulting in asymmetric curves. Epidemiologists concluded, thus, that the observed symmetry of the death curve in Wuhan was simply a contingent fact of Wuhan, instead of a regularity underlying the dynamics of the COVID-19 pandemic. Thus, a new version of the IHME model should avoid making that assumption with the goal of improving the accuracy of its estimates.

One of the aspects of the COVID-19 pandemic that became salient after the early predictions of the IHME model had been compared with the data was that people’s mobility (understood as the time they spent in public places) played a key role in shaping the mortality rate. Moreover, it also turned out that analogous political restrictions on mobility in different areas had strikingly different effects. This was partly because of the combination of different local conditions with people’s compliance with the introduced measures, which sharply varies in different geographical areas. For instance, different levels of population density in the area, differences concerning whether the measures are introduced in cities or in the countryside, or differences about the separation between residential and non-residential areas had a substantial impact on the outcome of the measures (IHME, 2020). This illustrates how hard it is for epidemiologists to make inferences based on data from a scarce pool of available locations, given the important magnitude of local effects.

**Fig. 9:**
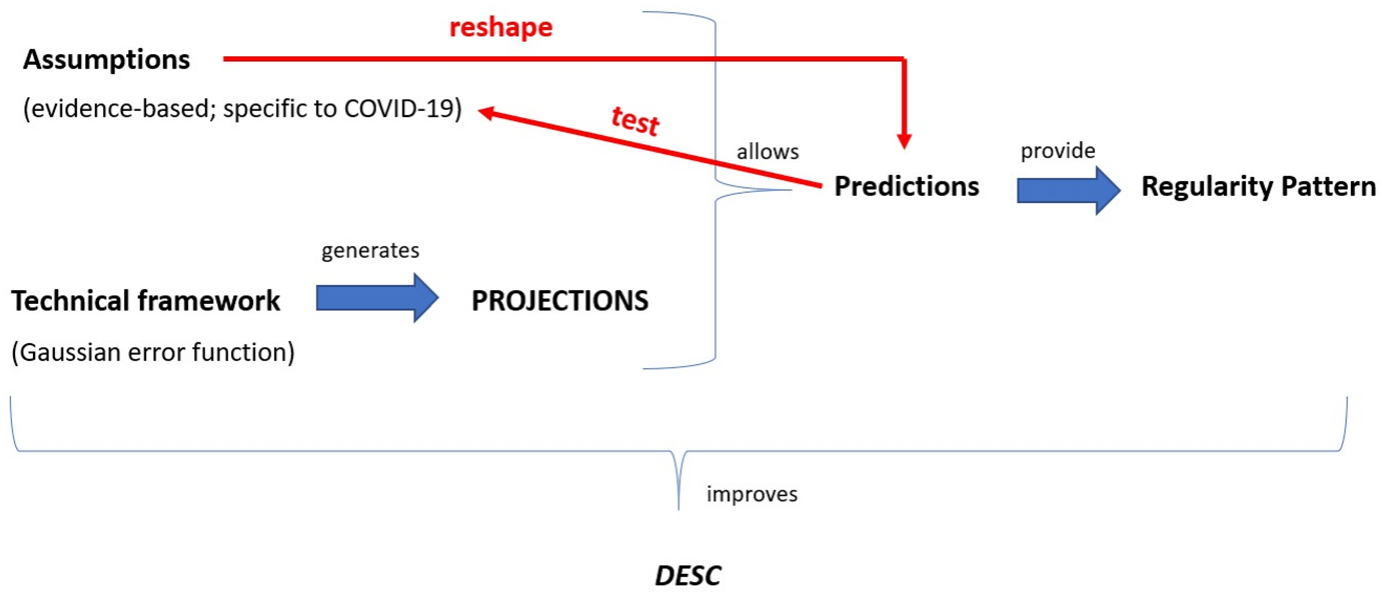
Schematic representation of the role of predictions in updating the IHME model. Notice that as the relationship between the predictions and the assumptions works back and forth, it is dialectical. Also note that, coherently with our claims, *DESC* gradually improves during the process of model update

We will now substantiate the claim that predictions play another fundamental epistemic role in the process leading to the obtainment of *DESC*. When comparing predictions with the actual evidence, they serve to *test* which of the assumptions that were used in building the model were mistaken. Note that the idea that predictions allow testing of the validity of the assumptions was already explicitly stated in the work of Douglas (2009) and de Regt (2017), yet they had failed to spell out why this fact played a role in the generation of scientific understanding, due to their emphasis on linking both concepts through an explanation (Sect. 2). Our hypothesis, as illustrated by the case of the IHME model, is that predictions help to generate descriptive understanding of a phenomenon by pinpointing the *error and success* of the model. That is, predictions play the additional role of showing where the model got the phenomenon right, and where it needs to be modified, so that deeper *DESC* can be gained. This generates a dialectical view of the relationship between *DESC* and prediction, one that we contend should be incorporated in contemporary analysis of the concepts, which is summarised when Fig. 8 is contrasted with Fig. 9. As this comparison illustrates, in the initial stage, the model generates predictions that provide a regularity pattern that allows scientists to obtain first degrees of *DESC* regarding the COVID-19- mortality rate. In the second stage, predictions work *backwards*: they are contrasted with the evidence and force scientists to reshape their model by modifying the assumptions upon which it relies. This second step does not require replacing the technical framework by a different one (i.e., by another type of error function), but merely updating it in the light of the introduction of some new assumptions that make the deduction of predictions feasible (by introducing so-called Gaussian extensions). Importantly, after this second stage has taken place, *the predictions are themselves altered*, i.e., they are freshly produced. This is because the change in the assumptions reshapes the model, hence the prediction generation process begins again, and in that vein, the whole *DESC*-prediction-assumption process is taken to the next stage, in which the assumptions and the predictions become more plausible for the phenomenon under investigation.^15^

Overall, this gives us a picture in which *DESC* and prediction are intimately linked in a dynamic way which results from the generation of a regularity pattern that is then compared with actual data. In the case of the IHME model, this was feasible, following the dynamics we have described, even in the early statistical versions published during March and April. This suggests that some statistical epidemiological models can provide scientific understanding of a special and non-explanatory type of certain phenomena, despite their lack of reference to causal or counterfactual variables affecting the dynamics of the phenomena. What makes understanding feasible in these cases is the model’s capacity for generating statistical associations between some key variables that underlie the phenomenon, even while it is not known how exactly these variables mechanistically relate to it. In other words, statistical models of COVID-19 such as the IHME model analysed here provide a genuine form of scientific understanding, which we have called *descriptive understanding* or *DESC*, even when the connection between the variables of the model and the real dynamics of the pandemic is not counterfactual.

In this vein, our work introduces a new modality of understanding that should be taken seriously in contemporary philosophical research. We have shown that prediction and understanding (or in de Regt’s terms *intelligibility*) of a model can also be linked via a description. We have shown that the degrees of *DESC* depends on the empirical appeal of the assumptions that allow the building of the model, its predictive capacity, and the possibility of modifying the assumptions in virtue of the predictions generated by the model.

## Conclusion

In an unprecedented manner, the COVID-19 pandemic caused rapidly growing rates of viral infections that threatened the lives of many and put hospitals all over the world in acute danger of becoming overwhelmed by the vast number of incoming patients. This emergency accelerated scientific research, with many resources being dedicated to understanding several aspects of the pandemic. One of the most important aspects to understand was the pandemic’s dynamics. Such dynamics includes uncovering how the transmission rate increases over time, how the mortality rate evolves, and how the number of infected people changes. To understand these aspects, scientists elaborated several epidemiological models. In this paper, we have studied one of these models, the IHME model, paying special attention to how it was modified and updated during the months of March and April 2020. These early versions of the IHME model followed a curve-fitting approach, and thus they were purely statistical as opposed to causal or causal-mechanistic models. Yet, they had *predictive capacity*, and their usefulness and relevance in political decision-making was based on this epistemic virtue.

In this paper, we have used the IHME model as a proxy to investigate how statistical epidemiological models can be scientifically useful, and what specific epistemic role they play in scientific research (Sect. 3). Our point of departure was the scientific conviction that these types of models provide scientific understanding of some phenomena (concretely, understanding of the dynamics of the COVID-19 mortality rate), and we have tried to uncover the reason why this family of epidemiological models is useful for this task. We have noticed that statistical models have substantial predictive power, and that this predictive power is intimately connected to their capacity in providing a regularity pattern for a concrete phenomenon. We have analysed the epistemological relationship between understanding and prediction on this basis. First, we have shown that statistical models do not provide explanatory understanding, contradicting some current accounts of understanding that equate it to *having an explanation*. We grounded our claim on the observation that statistical models do not spell out causal or counterfactual dependencies and, given that at least one of these should be present for a model to be considered explanatory, we have excluded this possibility (Sect. 4).

Second, we have coined the concept of *descriptive understanding* (*DESC*) which we characterised as the type of understanding that emerges and deepens in the process of building and modifying non-counterfactual, but plausible prediction-generating models or theories of the dynamics of a particular phenomenon. We defined the plausibility requirements in terms of the basic set of assumptions (A_1_, A_2_, A_3_, etc.) that scientists take to underlie the dynamics of the phenomenon based on what is known from previous research. In the specific case of the early versions of the IHME model, *DESC* is made feasible via the combination of a simple *technical framework* that depicts a mathematical function or regularity, with a series of *assumptions* about the set of variables that would affect the results of the technical framework and, in a sense, affect the unfolding of the phenomenon. The integration of these two aspects into a single model generates a set of predictions that, in turn, constitute a regularity pattern. *DESC*, we have argued, results from scientists’ ability to generate these regularity patterns and compare them with real data (Sect. 5; for a summary, see Fig. 8).

A second step in our work consisted in analysing how the original version of the IHME model changed in response to the growing evidence. This suggests that *DESC* is not a form of shallow understanding, but rather a genuine cognitive achievement, even though it comes in different degrees. We have shown that a key element that characterised the work of IHME modellers was that they used their predictions *backwards*, as instruments for comparing their model with the available evidence to gain knowledge about which assumptions of the model had to be changed. This step is fundamental, for it shows the dynamic interactions between predictions, DESC, and the assumptions that underlie model building, in a real-world case of model development. Our hypothesis was hence that predictions served scientists as a *test* to discover where their assumptions about the dynamics of COVID-19 had failed and needed to be replaced by new assumptions. Concretely, this means that certain local conditions not included in early versions of the IHME model (such as population density, urban area vs. countryside, or level of compliance with the mobility restrictions) needed to be included in an updated version, as they severely affected the development of COVID-19. In virtue of changing these assumptions, the whole prediction-generating process is started again (for a summary, see Fig. 9).

Our work emphasises an important step in the philosophical comprehension of scientific modelling (in its statistical version), scientific understanding, scientific prediction, and the relationship between them. We highlighted the epistemic value of non-causal, but *descriptive* models, and their relevance for contemporary scientific research. This suggests that understanding cannot merely consist in having an explanation (de Regt, 2017; Grimm, 2010; Hills, 2016; Khalifa, 2017), but it is a scientific skill that can be realised through multiple types of cognitive achievements (Dellsén, 2020; Verreault-Julien, 2019) (for a summary see Grimm et al., 2016; Grimm, 2021). Furthermore, we shed light on how these models evolve over time, and how they are generally responsive to evidence. An additional step would be to shed light on the role that these models have for the generation of hybrid or causal models (Sect. 3). As we said, after May 2020, the IHME became a hybrid model, as epidemiologists subsequently began using hybrid or causal models, instead of statistical models. Studying the role that early statistical models played in the development of hybrid models is, however, outside the scope of this work.

## Footnotes

1 The information in this and the next paragraph is a selective summary of: WHO’s Statement (27th April 2020) WHO Timeline—COVID-19”, World Health Organization, https://www.who.int/news/item/27-04-2020-who-timeline---covid-19. Accessed December 16, 2020. WHO’s Statement (29th June 2020) “Listings of WHO’s response to COVID-19″, *World Health Organization*. https://www.who.int/news/item/29-06-2020-covidtimeline. Accessed December 16, 2020.

2 Cornelius Hirsch (March 31, 2020) “Europe’s coronavirus lockdown measures compared”, *Politico*, https://www.politico.eu/article/europes-coronavirus-lockdown-measures-compared/. Accessed December 16,2020.

3 “Coronavirus: Field hospitals treating patients around world.” (March 30, 2020) *BBC News*, https://www.bbc.com/news/world-52089337. Accessed December 22, 2020.

4 William Booth, Christine Spolar, Pamela Rolfe (31st March 31, 2020) “Vast coronavirus ‘field hospitals’ fill spaces that hosted wedding expos and dog shows”. *The Washington Post*, https://www.washingtonpost.com/world/europe/coronavirus-field-hospitals/2020/03/31/3a05ba28-6f0f-11ea-a156-0048b62cdb51_story.html. Accessed December 22, 2020.

5 In the context of epidemiological modelling, statistical models are called *curve-fitting*, *data-fitting* and/or *data-driven* approaches indistinctly. In this paper, we will only use “curve-fitting” for parsimony.

6 By “purely predictive” we mean that the model only aims at producing a curve that fits the data available, rather than a model that is built on the basis of the causes of the phenomenon. See Sect. 2.

7 A note on the terminology: When we use the term *understanding* without any further specification concerning its type (explanatory, descriptive), it can be read as synonymous to what de Regt calls *intelligibility*. When we use *explanation* as the relationship that mediates between understanding and prediction, de Regt’s readers can interpret it as *explanatory understanding*. Our choice of this terminology is guided by the main purpose of the paper, which consists in distinguishing different modalities of understanding (explanatory/descriptive understanding), or different cognitive paths (explanation, description) that mediate between understanding (intelligibility) and prediction.

8 De Regt does not believe that CIT is the only criterion that exists to determine whether a theory is intelligible, but only *one criterion*. He leaves the question open as to whether there may be other possibilities.

9 Bear in mind intelligible theories exist in relation to a scientific community and the skill set of the scientists involved in working with the theory.

10 We should at this point recall that while we conveniently speak of *the* IHME model, the scientists who created the original model in March 2020 have since developed it in different versions in response to the acquisition of new empirical information. We have also shown that, through several updates, the core elements responsible for predicting the trajectories of the COVID-19 pandemic have been substantially altered, significantly increasing the accuracy of the predictive estimated. Thus, even if strictly speaking, there is not one IHME model, but a plurality of them, we will still speak of *the* IHME model for convenience.

11 Extracted from https://www.ncfp.org/2020/05/01/a-conversation-with-dr-chris-murray-of-the-institute-for-health-metrics-and-evaluation/. Accessed August 22, 2021.

12 We thank Carl Hoefer and an anonymous reviewer for raising this important point.

13 See “The criteria for Disease Outbreak News”. https://www.who.int/emergencies/disease-outbreak-news, Accessed December 22, 2020.

14 We are conscious that descriptions, and particularly these conveyed by drawings, play many other functions apart from conveying DESC, and the process of obtaining them presents unique challenges. But in our narrative, we are limiting the discussion to the cases where these descriptions and the process of generating them create DESC, as this is the main function that we characterize in this paper. We thank Hanna Worliczek for a very helpful discussion on this specific point.

15 One reviewer has objected that the fact that the assumptions can be isolated as the part that “went wrong” in the IHME model must be particular of this model, given confirmational holism, which causes that the theory or model is confirmed (or refuted) *as a whole* (Stanford 2017). This is essentially correct, but the reasons why IHME modellers though that the assumptions were what must have been wrong in this case depended on the fact that the choice of other parts of the model had been independently confirmed in other cases, e.g., for other diseases and in other outbreaks, whereas the assumptions where the key element that was being put into test given their locality (i.e., they were solely based on data from Wuhan, and some vague information, but had not received independent confirmation).
